# SARS-CoV-2 elicits non-sterilizing immunity and evades vaccine-induced immunity: implications for future vaccination strategies

**DOI:** 10.1007/s10654-023-00965-x

**Published:** 2023-02-04

**Authors:** Anna L. Beukenhorst, Clarissa M. Koch, Christoforos Hadjichrysanthou, Galit Alter, Frank de Wolf, Roy M. Anderson, Jaap Goudsmit

**Affiliations:** 1 Department of Biostatistics, Harvard T.H. Chan School of Public Health, Boston, MA USA; 2 Centre for Epidemiology Versus Arthritis, University of Manchester, Manchester Academic Health Science Centre, Manchester, UK; 3 Leyden Laboratories BV, Amsterdam, The Netherlands; 4 Department of Infectious Disease Epidemiology, School of Public Health, Imperial College London, London, UK; 5 Ragon Institute of MGH MIT and Harvard, Cambridge, MA USA; 6 Departments of Epidemiology, Immunology and Infectious Diseases, Harvard TH Chan School of Public Health, Boston, MA USA

**Keywords:** Respiratory infections, SARS-CoV-2 pandemic, COVID-19, Influenza, Vaccines, Vaccine effectiveness, Infection, Coronavirus, Immunology, Control measures, Herd immunity, Population modelling

## Abstract

Neither vaccination nor natural infection result in long-lasting protection against SARS-COV-2 infection and transmission, but both reduce the risk of severe COVID-19. To generate insights into optimal vaccination strategies for prevention of severe COVID-19 in the population, we extended a Susceptible-Exposed-Infectious-Removed (SEIR) mathematical model to compare the impact of vaccines that are highly protective against severe COVID-19 but not against infection and transmission, with those that block SARS-CoV-2 infection. Our analysis shows that vaccination strategies focusing on the prevention of severe COVID-19 are more effective than those focusing on creating of herd immunity. Key uncertainties that would affect the choice of vaccination strategies are: (1) the duration of protection against severe disease, (2) the protection against severe disease from variants that escape vaccine-induced immunity, (3) the incidence of long-COVID and level of protection provided by the vaccine, and (4) the rate of serious adverse events following vaccination, stratified by demographic variables.

## Introduction

Since the onset of the COVID-19 pandemic in 2020, it has become clear that neither infection nor the current vaccines [1–3] generate long-term immunity against SARS-CoV-2, because of rapidly waning antibody titres, continued viral evolution and high transmission rates [1, 4–8]. The transition of SARS-CoV-2 from a pandemic to an endemic state warrants reconsideration of future vaccination strategies, based on current evidence and the key uncertainties in the biology, epidemiology and evolution of the virus that remain.

## Sustained protection against severe disease

Emergency use vaccines against SARS-CoV-2 have substantially reduced rates of morbidity and mortality from COVID-19 in countries with high vaccination uptake, especially in high-risk groups such as the elderly. Observational studies in Israel, the UK, the United States and Qatar clearly show that vaccines provide robust protection against severe disease and death [9–13]. This may be due to cellular immunity since vaccine-induced memory B cells or T cells do not decline as rapidly as neutralizing antibody titers wane [3, 14, 15]. In addition, the recent Omicron variants have an upper respiratory tract tropism [16], which is associated with lower pathogenicity [17, 18], reduced risk of hospitalization [19, 20], but increased transmissibility.

## Reinfections due to rapid waning of immunity and immune escape

Protection against infection wanes rapidly both after vaccination [21] and natural infection [22]. Reinfections in vaccinated individuals and previously infected individuals have been common, especially with the Omicron variants [10, 23, 24].

This pattern of short-lasting immunity is similar to that of common cold viruses HCoV-NL63, HCoV-HKU1, HCoV-229E and HCoV-OC43 but different from the long-lasting immunity induced by SARS-CoV-1 and MERS [25, 26]. Antibodies elicited after SARS-CoV-2 infection and vaccination predominantly target the receptor-binding domain, which rapidly mutates [27, 28]. Through evolution, the virus can escape binding and neutralization by antibodies elicited after natural infection [29], and vaccination [11, 30–35]. Recent Omicron variants also escape therapeutic monoclonal antibodies [36–40].

Reinfections with SARS-CoV-2 reduce the risk of severe COVID-19 outcomes, in both vaccinated and in unvaccinated individuals [41, 42] and hospitalisation and death rates are lower in those who had a previous infection compared to those with a primary infection [9, 43].

## Community-wide control after transition from pandemic to endemic state

SARS-CoV-2 is on a pandemic-to-endemic trajectory with a strong seasonal transmission component. The long-term epidemiological pattern will be endemic persistence with peaks in transmission during the winter months plus recurrent surges of infection due to the emergence of novel variants [2, 44]. There may be multi-year period troughs and peaks in incidence (longer than seasonal outbreaks) determined by transmission intensity (the magnitude of the basic reproductive number R_0_ and the generation time of infection) and a population’s net birth rate (replenishing the supply of susceptible people) [2, 44, 45].

**Fig. 1:**
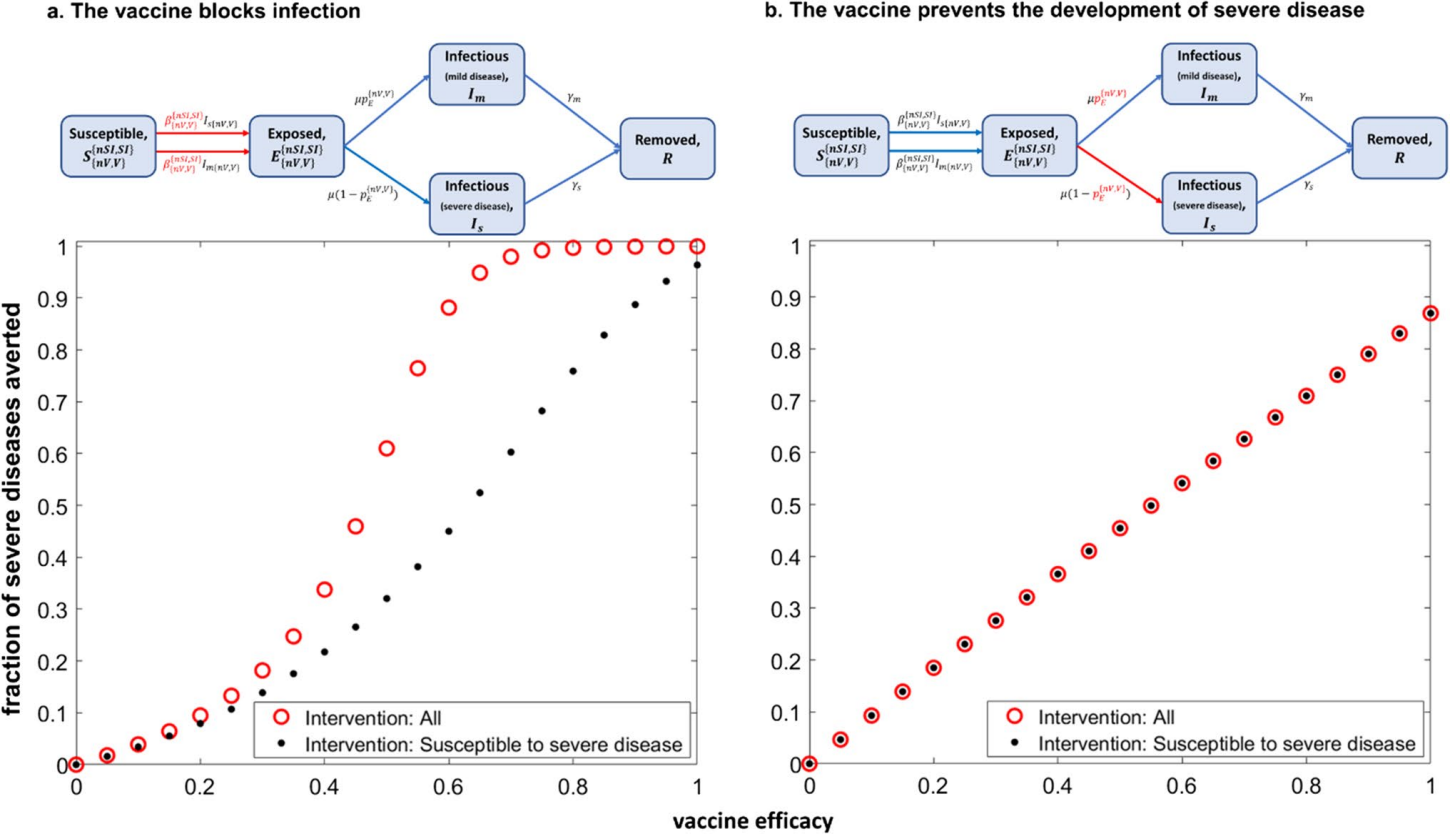
Comparison of ‘fraction of severe diseases averted’ as a function of vaccine efficacy between two vaccination strategies for **a** vaccine that blocks infection, and **b** vaccine that blocks severe disease. ﻿The classic Susceptible-Exposed-Infectious-Removed (SEIR) mathematical model was extended to describe the potential impact of vaccines of different mechanisms of action to generate insights into the corresponding optimal vaccination strategy for the prevention of severe diseases in the population [49, 50]. The schematic diagram shown displays all the transitions between the different infection states. All individuals are initially susceptible to infection. 33% of the total population is considered to be ‘susceptible to severe disease’ (SI), i.e., at a higher risk of developing severe disease (≥ 60 years old and those with comorbidities [51]). The rest of the population (nSI) is more likely to develop mild/asymptomatic disease. In this example, we consider the extreme scenario where the probability of the exposed SI group developing severe disease is equal to 1, whereas that of the nSI group is zero. Individuals in each group can be vaccinated (V) or not (nV). The output that is illustrated in the figures is the proportion of severe diseases averted within the first 120 days, for varying vaccine efficacy. The case where all individuals are vaccinated independently of whether they are susceptible to severe disease or not (‘All’) is compared to the case where only individuals that are susceptible to severe disease are vaccinated (‘Susceptible to severe disease’). The vaccination coverage is 85% in the SI group and 50% in the nSI group [51]. The model has been parametrised to describe the spread of SARS-CoV-2 in the early stages of the pandemic when R_0_ is low to moderate in value [50, 52]. ${R}_{0}$ = 2.8, latent period = 3.4 days

The transition from a pandemic to an endemic state has consequences for public health policy to moderate the impact of COVID-19, and especially the vaccination strategy to employ in the general population. Should this be part of childhood vaccination, should it be for the elderly and at risk only, or should the whole population be encouraged to get vaccinated? In cases where vaccines block infection, population-wide vaccination creates high levels of immunity, and has an advantage over vaccinating only those susceptible to severe disease (Fig. 1a). When immunity from vaccination is long-lasting, susceptible people are protected directly by vaccination or infection, and indirectly because transmission by others is less likely [46]. Implicitly, it was an expectation that the vaccines would protect against infection and transmission, as well as against severe disease. However, the growing body of evidence that vaccine protection against infection and transmission is so short-lived for SARS-CoV-2 indicates that population immunity would require vaccinating everybody at frequent intervals [47, 48].

With this combination of short-lived protection against infection and sustained protection against severe disease, little benefit arises from trying to create high levels of herd immunity even when vaccine efficacy is very high (> 90%) [1] (Fig. 1b﻿). A strategy of ‘vaccinating all’ is then less efficient, as it results in the same fraction of severe diseases averted as with the strategy of vaccinating only those susceptible to severe disease.

## Learning from the yearly influenza vaccination campaign

For influenza, the yearly vaccination also elicits non-sterilizing immunity due to continued viral evolution. Vaccinating the vulnerable and those in high-risk environments reduces hospitalizations and deaths, even at reasonably low coverage [53]. However, the R_0_ for influenza A is low: <1 in the summer season in the northern hemisphere and around 1.28 in winter, enabling effective herd immunity at lower coverages [54]. For SARS-CoV-2, herd immunity would not be achievable, even at high coverage, given its much higher R_0_ value (> 5 for some Omicron strains). Although risk groups for severe influenza and SARS-CoV-2 partially overlap, coadministration of SARS-CoV-2 vaccines with the yearly flu jab is logistically challenging. Many of the current vaccines are not syringe-filled and have challenging temperature storage requirements, hampering fast administration at GPs and pharmacies.

## Key uncertainties

The best vaccination strategy – balancing health benefits with cost of implementation – will be different for the endemic SARS-CoV-2 compared to the pandemic SARS-CoV-2. If past vaccination continues to offer some strong protection against severe disease and associated mortality, then future vaccination strategies could be selective rather than community-wide.

Various uncertainties hamper weighing the benefits and disadvantages of vaccination. First, the long-term protection against disease by vaccination or natural infection is unknown, as we are only at year 2 since the start of mass immunisation campaigns. Vaccinating risk groups only may be sufficient as long as  non-risk groups are still protected from severe disease by previous infections and past vaccination. Second, little data is available on the incidence of morbidity due to long COVID and reduction of that incidence by vaccines. Long COVID seems to occur mainly after otherwise mild infections, and in people who do not have an increased risk of hospitalization or death [55–57]. In young age groups, early experience of SARS-CoV-2 could be beneficial, but only if the incidence of long COVID in these groups is low compared to the rate of serious adverse events after [58]. Third, high quality data on the rate of serious adverse events after vaccination, and the risk of severe disease following reinfection should be collected as an urgent priority, stratified by gender, age group, and various comorbidities including immunosuppression. Furthermore, the risk of severe disease following reinfection will change as SARS-CoV-2 continues to evolve worldwide in directions that are difficult to predict. Evolution will drive to increased transmissibility but this may or may not be linked to reduced or increased pathogenicity [59]. Variants less well-matched to the strains used for current vaccines may escape prior immunity unless vaccines are updated to the dominant circulating strains.

## Implications for future vaccination strategies

To resolve key uncertainties, phase IV studies are needed on the following topics carefully stratified by a series of demographic variables such as age, sex, comorbidities and vaccination status: (1) the duration of protection against severe disease; (2) the level of protection against severe disease from well-matched versus differing variants; (3) the incidence of long COVID; and (4) the rate of serious adverse events following vaccination.

Such information should inform governments considering moving to a vaccination strategy that centres on morbidity and mortality prevention, instead of transmission control via the creation of herd immunity. Groups at high risk for mortality and hospital admissions should then be prioritized [48]. Although precise identification of the most vulnerable is challenging, various factors, including age and certain comorbidities, are clearly associated with increased risk of severe COVID-19. Further data on progressing to long COVID is needed, especially in the younger age groups that otherwise have lower risks of severe disease [55, 56].

Worldwide, the evolution of SARS-CoV-2 should be tracked to warn about the emergence of new strains that escape immunity, or have enhanced transmissibility and pathogenicity [59]. Improving the durability of immunity across strains should be a research priority, for example through development of multivalent vaccines as in the case of the pneumococcal vaccines. In future, the non-sterilizing immunity conferred by vaccines in those at risk could be combined with novel antiviral therapies that prevent infection, lower viral load and concomitantly lower transmissibility and pathogenicity.

## References

[1] Anderson RM (2021). The SARS-CoV-2 pandemic: remaining uncertainties in our understanding of the epidemiology and transmission dynamics of the virus, and challenges to be overcome. Interface Focus.

[2] Mullin S, van der Wyk B, Asher JL, Compton SR, Allore HG, Zeiss CJ (2022). Modeling pandemic to endemic patterns of SARS-CoV-2 transmission using parameters estimated from animal model data. PNAS Nexus.

[3] Barouch DH (2022). Covid-19 vaccines—immunity, variants, boosters. N Engl J Med.

[4] Singanayagam A (2022). Community transmission and viral load kinetics of the SARS-CoV-2 delta (B.1.617.2) variant in vaccinated and unvaccinated individuals in the UK: a prospective, longitudinal, cohort study. Lancet Infect Dis.

[5] Allen H (2022). Comparative transmission of SARS-CoV-2 Omicron (B.1.1.529) and Delta (B.1.617.2) variants and the impact of vaccination: national cohort study, England. MedRxiv.

[6] Sheward, Daniel J., et al. “Evasion of neutralising antibodies by omicron sublineage BA. 2.75.” The Lancet Infectious Diseases 22.10 (2022): 1421–1422. 10.1016/S1473-3099(22)00524-2 10.1016/S1473-3099(22)00524-2 PMC9436366 36058228

[7] Cao Y (2022). Imprinted SARS-CoV-2 humoral immunity induces convergent Omicron RBD evolution. Nature.

[8] Altarawneh HN (2022). Protective Effect of Previous SARS-CoV-2 Infection against Omicron BA.4 and BA.5 Subvariants. N Engl J Med.

[9] Agrawal U (2022). Severe COVID-19 outcomes after full vaccination of primary schedule and initial boosters: pooled analysis of national prospective cohort studies of 30 million individuals in England, Northern Ireland, Scotland, and Wales. The Lancet.

[10] Zheutlin Amanda, et al. Durability of Protection Post–Primary COVID-19 Vaccination in the United States. Vaccines 10.9 (2022): 1458. https://www.mdpi.com/2076-393X/10/9/1458 10.3390/vaccines10091458 PMC9505933 36146536

[11] Abu-Raddad LJ, Chemaitelly H, Bertollini R (2022). Waning mRNA-1273 Vaccine Effectiveness against SARS-CoV-2 Infection in Qatar. N Engl J Med.

[12] Chemaitelly H (2021). Waning of BNT162b2 Vaccine Protection against SARS-CoV-2 Infection in Qatar. N Engl J Med.

[13] Goldberg Y (2021). Waning immunity after the BNT162b2 vaccine in Israel. N Engl J Med..

[14] Sokal A (2022). Analysis of mRNA vaccination-elicited RBD-specific memory B cells reveals strong but incomplete immune escape of the SARS-CoV-2 Omicron variant. Immun Apr.

[15] Tarke A (2022). SARS-CoV-2 vaccination induces immunological T cell memory able to cross-recognize variants from Alpha to Omicron. Cell.

[16] Meng B (2022). Altered TMPRSS2 usage by SARS-CoV-2 omicron impacts infectivity and fusogenicity. Nature..

[17] McMahan K (2022). Reduced pathogenicity of the SARS-CoV-2 omicron variant in hamsters. Med.

[18] Armando F (2022). SARS-CoV-2 Omicron variant causes mild pathology in the upper and lower respiratory tract of hamsters. Nat Commun..

[19] Bager P (2022). Risk of hospitalisation associated with infection with SARS-CoV-2 omicron variant versus delta variant in Denmark: an observational cohort study. Lancet Infect Dis..

[20] Veneti L (2022). Reduced risk of hospitalisation among reported COVID-19 cases infected with the SARS-CoV-2 Omicron BA.1 variant compared with the Delta variant, Norway, December 2021 to January 2022. Eurosurveillance.

[21] Altarawneh HN (2022). Effects of previous infection and vaccination on symptomatic omicron infections. N Engl J Med.

[22] Spicer KB, Glick C, Cavanaugh AM, Thoroughman D (2020). Protective immunity after natural infection with severe acute respiratory syndrome coronavirus-2 (SARS-CoV-2)–Kentucky, USA, 2020. Int J Infect Dis.

[23] Roskosky M (2022). Notes from the field:SARS-CoV-2 omicron variant infection in 10 persons within 90 days of previous SARS-CoV-2 delta variant infection—Four States, October 2021–January 2022. MMWR Morb Mortal Wkly Rep.

[24] Pulliam JRC (2022). Increased risk of SARS-CoV-2 reinfection associated with emergence of Omicron in South Africa. Science (1979).

[25] Edridge AWD (2020). Seasonal coronavirus protective immunity is short-lasting. Nat Med.

[26] Mwita Morobe J (2021). Trends and Intensity of Rhinovirus Invasions in Kilifi, Coastal Kenya, Over a 12-Year Period, 2007–2018. Open Forum Infect Dis.

[27] Mykytyn AZ (2022). Antigenic cartography of SARS-CoV-2 reveals that Omicron BA. 1 and BA. 2 are antigenically distinct.. Science Immunology.

[28] Kumar S, Karuppanan K, Subramaniam G (2022). Omicron. (BA.1) and sub-variants (BA.1, BA.2 and BA.3) of SARS-CoV-2 spike infectivity and pathogenicity: a comparative sequence and structural-based computational assessment. J Med Virol.

[29] Garcia-Valtanen P (2022). SARS-CoV-2 Omicron variant escapes neutralizing antibodies and T cell responses more efficiently than other variants in mild COVID-19 convalescents. Cell Rep Med..

[30] Arora P (2022). SARS-CoV-2 Omicron sublineages show comparable cell entry but differential neutralization by therapeutic antibodies. Cell Host Microbe.

[31] Iketani S (2022). Antibody evasion properties of SARS-CoV-2 Omicron sublineages. Nature..

[32] Zhou H, Dcosta BM, Landau NR, Tada T (2022). Resistance of SARS-CoV-2 Omicron BA.1 and BA.2 variants to vaccine-elicited sera and therapeutic monoclonal antibodies. Viruses.

[33] Wang Q (2022). Antibody evasion by SARS-CoV-2 Omicron subvariants BA.2.12.1, BA.4, and BA.5. Nature.

[34] Quandt J (2022). Omicron BA.1 breakthrough infection drives cross-variant neutralization and memory B cell formation against conserved epitopes. Sci Immunol.

[35] Alter G (2021). Immunogenicity of Ad26.COV2.S vaccine against SARS-CoV-2 variants in humans. Nature.

[36] Arora P (2022). Augmented neutralisation resistance of emerging omicron subvariants BA.2.12.1, BA.4, and BA.5. Lancet Infect Dis.

[37] Cele S (2022). Omicron extensively but incompletely escapes Pfizer BNT162b2 neutralization. Nature..

[38] Hachmann NP (2022). Neutralization escape by SARS-CoV-2 omicron subvariants BA.2.12.1, BA.4, and BA.5. N Engl J Med.

[39] Lassaunière R (2022). Neutralizing antibodies against the SARS-CoV-2 omicron variant (BA.1) 1 to 18 weeks after the second and third doses of the BNT162b2 mRNA vaccine. JAMA Netw Open.

[40] van Gils MJ (2022). Antibody responses against SARS-CoV-2 variants induced by four different SARS-CoV-2 vaccines in health care workers in the Netherlands: a prospective cohort study. PLoS Med..

[41] Medić S (2022). Risk and severity of SARS-CoV-2 reinfections during 2020–2022 in Vojvodina, Serbia: a population-level observational study. Lancet Reg Health Eur.

[42] Yahav D (2021). Definitions for coronavirus disease 2019 reinfection, relapse and PCR re-positivity. Clin Microbiol Infect..

[43] Michlmayr D (2022). Observed protection against SARS-CoV-2 reinfection following a primary infection: a Danish cohort study among unvaccinated using two years of nationwide PCR-test data. Lancet Reg Health Eur.

[44] Kissler SM, Tedijanto C, Goldstein E, Grad YH, Lipsitch M (2020). Projecting the transmission dynamics of SARS-CoV-2 through the postpandemic period. Science (1979).

[45] Anderson RM, May RM (1992). Infectious diseases of humans: dynamics and control.

[46] Anderson RM, May RM (1985). Vaccination and herd immunity to infectious diseases. Nature.

[47] Feikin DR (2022). Duration of effectiveness of vaccines against SARS-CoV-2 infection and COVID-19 disease: results of a systematic review and meta-regression. The Lancet.

[48] Anderson RM, Vegvari C, Truscott J, Collyer BS (2020). Challenges in creating herd immunity to SARS-CoV-2 infection by mass vaccination. The Lancet.

[49] Walker PGT (2020). The impact of COVID-19 and strategies for mitigation and suppression in low- and middle-income countries. Science (1979).

[50] Hadjichrysanthou C (2022). Exploring the role of antiviral nasal sprays in the control of emerging respiratory infections in the community. Infect Dis Ther.

[51] Kelly SL, le Rutte EA, Richter M, Penny MA, Shattock AJ (2022). COVID-19 vaccine booster strategies in light of emerging viral variants: frequency, timing, and target groups. Infect Dis Ther.

[52] Knock ES (2021). Key epidemiological drivers and impact of interventions in the 2020 SARS-CoV-2 epidemic in Englan. Sci Transl Med.

[53] Backer JA, Wallinga J, Meijer A, Donker GA, van der Hoek W, van Boven M (2019). The impact of influenza vaccination on infection, hospitalisation and mortality in the Netherlands between 2003 and 2015. Epidemics.

[54] Biggerstaff M, Cauchemez S, Reed C, Gambhir M, Finelli L (2014). Estimates of the reproduction number for seasonal, pandemic, and zoonotic influenza: a systematic review of the literature. BMC Infect Dis.

[55] Zimmermann P, Pittet LF, Curtis N (2021). How common is long COVID in children and adolescents?. Pediatr Infect Dis J.

[56] Crook H, Raza S, Nowell J, Young M, Edison P (2021). Long covid—mechanisms, risk factors, and management. BMJ.

[57] Brito AF (2022). Global disparities in SARS-CoV-2 genomic surveillance. Nat Commun..

[58] Davis, Hannah E., et al. “Long COVID: major findings, mechanisms and recommendations.” Nature Reviews Microbiology (2023): 1–14. 10.1038/s41579-022-00846-2 PMC9839201 36639608

[59] Anderson RM, May RM (1982). Coevolution of hosts and parasites. Parasitology.

